# Biomarkers in Acute Myeloid Leukemia: Leveraging Next Generation Sequencing Data for Optimal Therapeutic Strategies

**DOI:** 10.3389/fonc.2021.748250

**Published:** 2021-09-30

**Authors:** Hanadi El Achi, Rashmi Kanagal-Shamanna

**Affiliations:** Department of Hematopathology, The University of Texas MD Anderson Cancer Center, Houston, TX, United States

**Keywords:** AML, acute myeloid leukemia, next generation sequencing, actionable mutations, targeted therapy, FDA

## Abstract

Next generation sequencing (NGS) is routinely used for mutation profiling of acute myeloid leukemia. The extensive application of NGS in hematologic malignancies, and its significant association with the outcomes in multiple large cohorts constituted a proof of concept that AML phenotype is driven by underlying mutational signature and is amenable for targeted therapies. These findings urged incorporation of molecular results into the latest World Health Organization (WHO) sub-classification and integration into risk-stratification and treatment guidelines by the European Leukemia Net. NGS mutation profiling provides a large amount of information that guides diagnosis and management, dependent on the type and number of gene mutations, variant allele frequency and amenability to targeted therapeutics. Hence, molecular mutational profiling is an integral component for work-up of AML and multiple leukemic entities. In addition, there is a vast amount of informative data that can be obtained from routine clinical NGS sequencing beyond diagnosis, prognostication and therapeutic targeting. These include identification of evidence regarding the ontogeny of the disease, underlying germline predisposition and clonal hematopoiesis, serial monitoring to assess the effectiveness of therapy and resistance mutations, which have broader implications for management. In this review, using a few prototypic genes in AML, we will summarize the clinical applications of NGS generated data for optimal AML management, with emphasis on the recently described entities and Food and Drug Administration approved target therapies.

## Introduction

**Figure 1:**
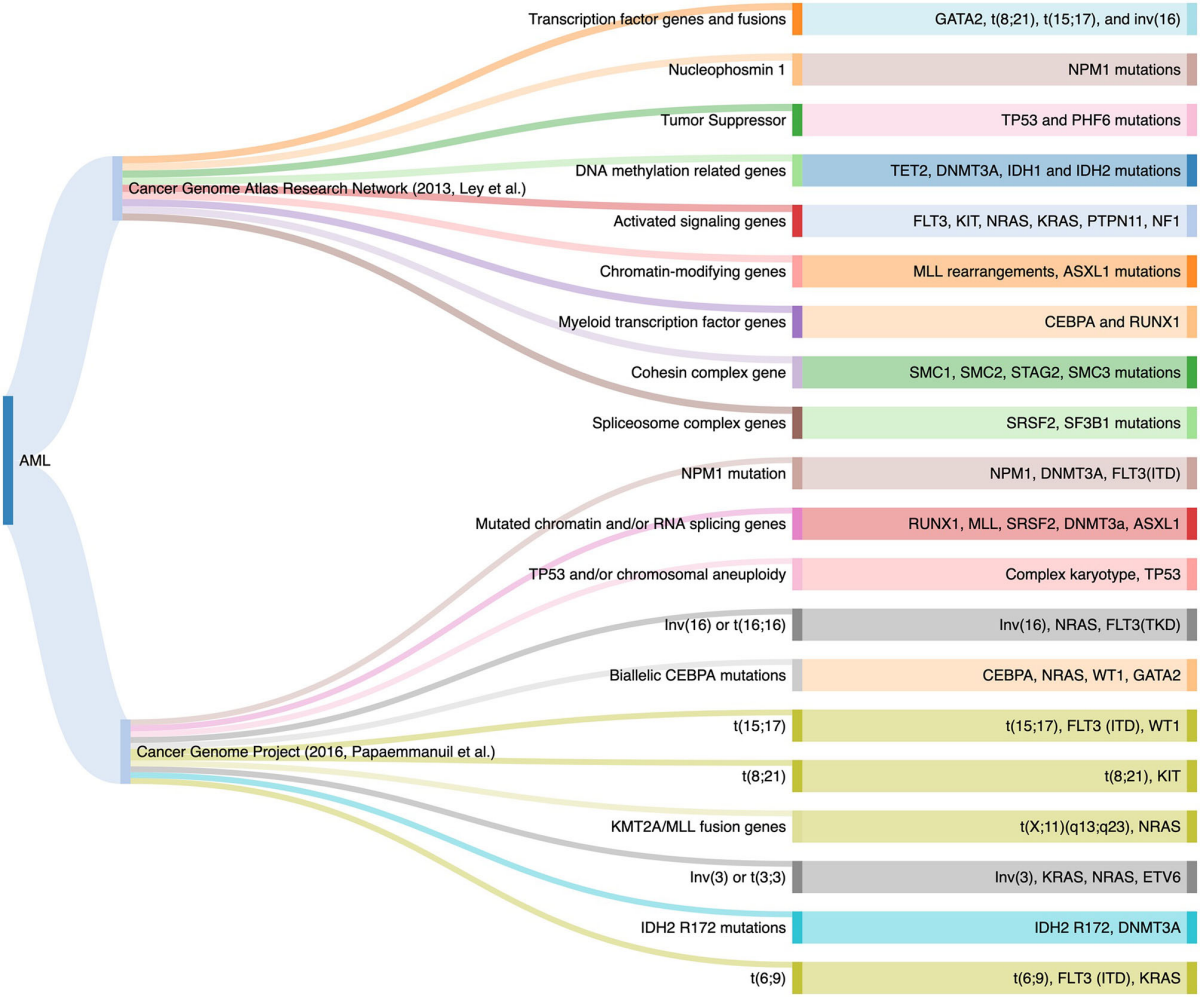
Genomic Classifications of Acute Myeloid Leukemia as Proposed by the TCGA Project and the 2016 Study.

Acute myeloid leukemia (1) is a clonal malignant expansion of immature myeloid precursors due to block in differentiation. Mutation profiling is standard for routine baseline clinical evaluation of AML. Prior to 2008, there were two functional genetic groups for leukemic pathogenesis: class I (activated signaling genes such as *FLT3*, *KIT* and *RAS* mutations) that conferred the proliferative potential, and class II genes involved in transcription and differentiation such as *CEBPA* and *RUNX1* (2). The development of high-throughput NGS sequencing platforms uncovered many somatic mutations in AML. In 2013, the Cancer Genome Atlas (TCGA) project expanded the functional genetic classes to nine families involved in the pathogenesis of the myeloid neoplasms. An average of 14 mutations were identified in the AML genome, ranging between five to 23 genetic mutations in each case (3). In 2016, a large cohort that enrolled 1540 AML patients described 11 subgroups of genomic alterations with different clinical outcomes using NGS. The additional separate categories included AML with mutations in genes encoding chromatin and RNA-splicing regulators, AML with *TP53* mutations and/or chromosomal aneuploidies, and AML with *IDH2* R172 mutations (4). Taken together, these findings supported the fact that AML phenotype is driven by underlying mutational signature (**Figure 1**).

Accordingly, both the 2016 revisions to the WHO and 2017 European LeukemiaNet (5) incorporated gene mutations into the sub-classification and risk-stratification of AML (6, 7). Molecular testing plays a major role in the current World Health Organization (WHO) classification of myeloid neoplasms. Specific genetic abnormalities are regarded as disease-defining mutations and others have important prognostic and therapeutic implications. The WHO recognizes 3 distinct sub-categories of AML based on somatic mutations: AML with *NPM1* mutation, AML with bi-allelic mutations of *CEBPA*, and AML with mutated *RUNX1* (provisional entity). Genes such as *TP53, RUNX1, IDH1/2* and FMS-related tyrosine kinase 3 (8), among others, are found to be altered in the different subcategories of AML with prognostic and/or therapeutic implications of utmost importance. The latest 2017 European Leukemia Net (ENL) guidelines for AML recommends molecular profiling for mutations in *NPM1, CEBPA, FLT3-ITD, TP53, RUNX1, ASXL1*, and *BCR-ABL1.* Therefore, the complete cytogenetic and molecular work-up is essential at the time of initial AML evaluation. The main goal of genome sequencing is the identification of actionable gene mutations and risk-stratification. Hence, multi-gene panel next-generation sequencing (NGS) based mutation analysis is the primary mode for assessment for multiple AML associated genes (9).

In this review, we will provide an overview of the diagnostic, prognostic and therapeutic data that can be obtained from routine clinical NGS data in AML. In addition, we will describe the implications of the vast amount of additional data that can be obtained from routine clinical NGS beyond prognostication and management, such as the ontogeny of AML, clues regarding underlying germline predisposition and clonal hematopoiesis, serial monitoring to assess the effectiveness of therapy and resistance mutations. We will use a few prototypic genes for each section, with emphasis on the recently described entities and actionable mutations, specifically pertaining to the Food and Drug Administration (10) approved target therapies and the ongoing clinical trials. AML-defining translocations are beyond the scope of this review.

## Genetic Alterations With FDA-Approved Targeted Therapies

### AML With Mutated *FLT3*

**Category:** activated signaling genes.

**Clinicopathologic and morphologic features:** Leukocytosis.

**Recommended method of testing:** NGS for tyrosine kinase domain (TKD) mutations (25) and small internal tandem duplications (8), Polymerase Chain Reaction (PCR) followed by capillary electrophoresis (CE) for medium and large internal tandem duplications (8, 31).

*FLT3* is a receptor with tyrosine kinase (TK) activity, involved in proliferation and differentiation of hematopoietic progenitors. The associated mutations generally affect the juxta-membrane and the TK domain of the receptor. Broadly, mutations in *FLT3* are of two types: *FLT3*-ITD which is the most frequent alteration, and *FLT3*-TKD which consists of a point mutation in the TK domain (25).

#### *FLT3*-ITD

*FLT3*-ITD are in-frame mutations which consist of duplication of small sequences, ranging from 3 to larger than 400 base pairs resulting in a receptor with an elongated juxta-membrane domain. This leads to constitutive activation of the receptor and activation of intracellular pathways resulting in cellular proliferation (25, 32). The frequency of *FLT3*-ITD mutations in AML ranges from 20-50% (30). *FLT3*-ITD is associated with proliferative AML with a high WBC count (25). The prognostic implication of the mutation is dependent on the allelic burden (33). The allele burden of *FLT3*-ITD can be measured using one of the two parameters (10): allele ratio (AR), defined as the ratio of the area under the curve of mutant to wild-type and (2) allele frequency (AF) which is defined as the ratio of the area under the curve of mutant to total (mutant + wild-type). The 2017 ELN adopts a cut-off of 0.5 to differentiate between low *versus* high AR (34). AML patients with high AR *FLT3*-ITD had significantly low complete remission (CR) rates, with poor survival and relapse; only high *FLT3*-ITD AR patients (≥0.5) benefited from allogeneic stem cell transplantation (32). Per ELN, *NPM1* mutated AML with *FLT3*-ITD AR <0.5 is considered as a favorable prognostic subgroup, similar to AML with absent *FLT3*-ITD mutation, and stem cell transplant is not recommended (34).

#### *FLT3*-TKD

*FLT3*-TKD mutations are less common than the *FLT3*-ITD; they consist mainly of missense point mutations, deletions or insertions within the TK domain. The most frequent alteration is a point mutation involving nucleotide substitution on codon 835. NGS can identify numerous mutations outside of D835, including deletions. The definitive implications of these various *FLT3*-TKD in prognostic stratification are still under review (25).

*FLT3* mutations can be targeted using tyrosine kinase inhibitor drugs in combination with standard chemotherapy. The addition of tyrosine kinase inhibitor (TKI), Midostaurin, to standard chemotherapy protocol for AML led to significantly longer overall survival (OS) and event-free survival (EFS) in three *FLT3* subgroups: *FLT3*-TKD, *FLT3*-ITD low and *FLT3*-ITD high AR (RATIFY-NCT00651261) (12). Midostaurin, the first FDA approved targeted therapy in AML, is a non-selective first generation TKI that targets multiple other pathways including c-KIT, PKC, PDGFR, VEGFR, resulting in higher toxicity (13). In 2018, a more selective “second-generation” *FLT3* inhibitor, Gilteritinib, with fewer side-effects received FDA approval for relapsed or refractory *FLT3*-ITD or *FLT3*-TKD mutations-positive AML (14) based on the results of ADMIRAL (NCT02421939) and CHRYSALIS (NCT02014558) clinical trials, both of which demonstrated significantly improved outcomes in the Gilteritinib group (15, 16). Other first and second generation of inhibitors for *FLT3* such as Sorafenib (NCT01398501) (31) and Quizartinib (NCT02668653) (in newly diagnosed AML) (8), have currently reached late stages of clinical testing.

While NGS is ideal for identification of mutations across the entire coding region, specifically FLT3 TKD mutations and small FLT3 ITDs, amplicon-based targeted NGS is unable to pick up majority of the medium to larger ITDs. Hence, ITD mutations are always tested concurrently by PCR followed by fragment analysis using capillary electrophoresis (CE). Alternate computational algorithms, such as Pindel to analyze NGS data have shown promising results with 100% sensitivity and specificity in detecting medium and large insertions at 1% VAF (35). Pindel is a pattern growth algorithm to detect breakpoints of medium and large alterations from paired-end short reads (36).

**Figure 2:**
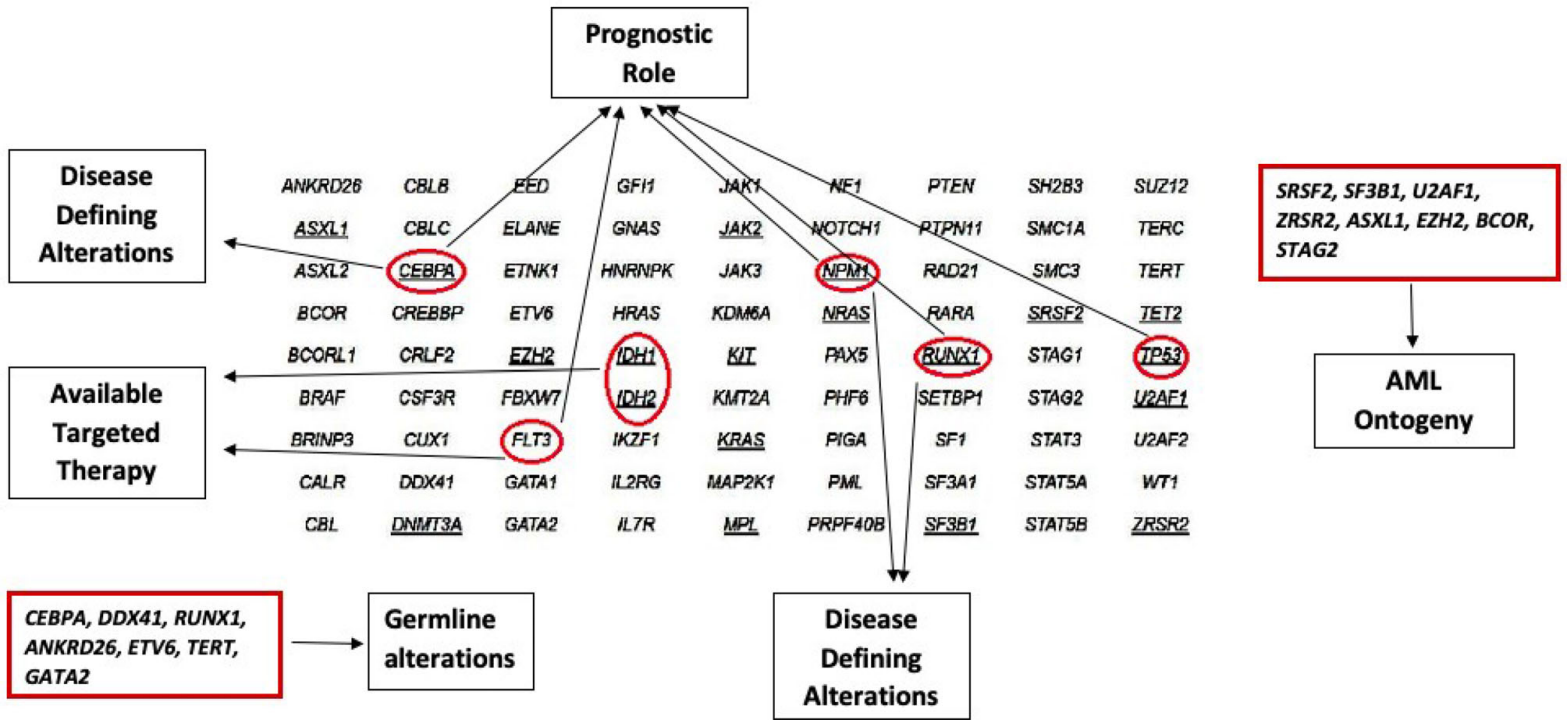
Role of different Genetic Alterations as Diagnostic, and Prognostic markers, as well as the Availability of Targeted Therapy.

Resistance mutations to *FLT3* inhibitors can emerge over the course of therapy *via* activation of alternative alternate intracellular pathways. Certain type II (second generation) *FLT3* inhibitors do not have activity against TKD mutations, therefore the emergence of a *FLT3*-TKD mutation during treatment, particularly *FLT3* D835 mutation would confer resistance to TKI (16). Crenolanib is a second generation inhibitor with activity against ITD and TKD mutations, hence, able to overcome the treatment resistance resulting from *FLT3*-TKD alteration (25). Other mechanisms of resistance to *FLT3* inhibitors include the emergence of leukemia clones harboring mutations that activate RAS/MAPK pathway signaling, or *BCR-ABL1* fusions (37). Hence, sequential mutation analysis is important for early detection of these resistant clone to modulate the therapy accordingly, prior to overt morphologic relapse (**Tables 1**, **2** and **Figure 2**).

**Table 1:** Summary of key FDA approved targeted therapy for acute myeloid leukemia treatment.

	Drug	Targeted gene/protein	Year of approval	Study/Clinical trial	Indication	Response Rate (RR) Complete Remission (CR) Overall Survival (OS)	Refs
1	**Enasidenib**	*IDH2*	2017	NCT01915498	Relapsed/refractory AML patients	40.3% RR 19.3% CR 5.8 months OS	11
2	**Ivosidenib**	*IDH1*	2019	NCT02074839	Relapsed/refractory AML	41.6% RR 21.6% CR 9.3 months OS	10
3	**Midostaurin**	*FLT3*	2017	RATIFY (NCT00651261)	Newly diagnosed AML with *FLT3*-TKD or *FLT3*-ITD mutations	58.9% CR 74.7 months OS	12, 13
4	**Gilteritinib**	*FLT3*	2018	ADMIRAL (NCT02421939)	Relapsed/refractory AML showing *FLT3*-TKD or *FLT3*-ITD mutations	34% CR 9.3 months OS	14–16
				CHRYSALIS (NCT02014558)		52% RR 41% CR 31 months OS	
5	**Quizartinib**	*FLT3*	2019	Approved only in Japan Study: NCT02668653	Relapsed/refractory AML showing *FLT3*-ITD mutations	48% CR 6.2 months OS	8
6	**Venetoclax**	BCL2 protein	2018	NCT02203773	Newly diagnosed AML in patients > 75 years old or who have severe comorbidities	37% CR with azacitidine 54% CR with decitabine	14, 17, 18
				NCT02287233		21% CR with cytarabine	

**Table 2:** Summary of distribution of frequencies of genes mutations in Acute Myeloid Leukemia.

Genetic Mutation	Frequency in AML (%)	Refs
*ETV6*	1	19
*KIT*	2	20
*DDX41*	3	21
*TERT*	3	22
*IDH1*	4-9	23
Biallelic *CEBPA*	4-9	24
*FLT3-*TKD	7-10	25
*IDH2*	8-19	23
*TP53* in *de novo* AML *TP53* in therapy related AML	8-14 60	26–28
*GATA2*	9	29
*RUNX1* (somatic)	4-16	24
*NPM1*	27	4
*FLT3-*ITD	20-50	30

### AML With Mutated *IDH1* and *IDH2*

**Category:** DNA methylation related.

**Clinicopathologic and morphologic features:** AML without maturation [French-American-British classification (FAB) M1], AML with maturation (FAB M2), and acute monocytic leukemia (FAB M5).

**Recommended method of testing:** NGS, droplet digital PCR (ddPCR) for MRD (38).

*IDH1* and *IDH2* are DNA methylation genes; the mutations in *IDH* induce dysregulation of epigenetic methylation particularly the function of the *TET* family of methylators. These aberrations will ultimately result in muting the pathways involved in differentiation of hematopoietic progenitors leading to maturation arrest (5). Moreover, *IDH* mutations diminish the DNA repair mechanism and result in the accumulation of secondary mutations (39). *IDH1* and *IDH2* mutations are associated with AML in 4–9% and 8–19% of the cases respectively; they are generally a founding clone sufficient to cause overt leukemia without additional genetic alterations (23). The hotspot mutations involve amino-acid substitutions at codon 132 in exon 4 of the *IDH1* gene and codons 140 or 172 in exon 4 of *IDH2*. *IDH2*-R172 is mutually exclusive with *NPM1* mutation and is regarded as an independent sub-category by genomic analysis, but not currently recognized by the WHO (40).

Similar to *FLT3*, mutations in *IDH1*/2 are a prototypic example of targeted therapy in AML. Enasidenib is an oral selective inhibitor of mutant *IDH2* enzyme variants R140Q, R172S, and R172K (11); it was FDA approved in 2017 for the treatment of relapsed/refractory AML patients (11). Ivosidenib targets mutant IDH1enzyme leading to normal differentiation and maturation of malignant cells, and was FDA approved in 2019 for treatment of relapsed/refractory AML cases (10).

Despite the presence of hotspot mutations, NGS is ideally suited for baseline identification, serial monitoring of response to therapy and relapse emergence. Droplet digital PCR for specific mutations during serial monitoring can provide a higher sensitivity than standard NGS, however, can miss detection new emerging mutations (**Tables 1**, **2** and **Figure 2**).

### AML With Mutated *KIT*

**Category:** Kinase signaling pathway.

**Clinicopathologic and morphologic features:**

Seen mostly in core-binding factor (CBF) leukemias that encompass AML with inv(16) and t(8;21). In the absence of either translocations, detection of KIT mutation is a helpful clue to search for underlying mastocytosis.

**Recommended method of testing:** 1-(10) Allele-specific PCR to detect exon 17 D816V specifically (41); 2- NGS (preferred) or other sequencing techniques covering for exons 17 and 8.

Activating mutations of *KIT* (encoding a transmembrane glycoprotein) leading to constitutional activation of receptor tyrosine kinase pathway, similar to systemic mastocytosis, GIST and germ cell tumors, are most commonly observed in “core binding factor” (CBF) leukemias which encompass AML with t(8;21) and AML with inv(16). Gain of function mutations in *KIT* have been found in 2% of AML overall and in a 33% of the CBF leukemias. These mutations tend to occur within exon 17, most importantly the hotspot D816, and exon 8 of the *KIT* gene (42).

Although CBF-AML has a favorable overall prognosis, most reports indicate that co-occurrence of *KIT* mutations confer an adverse prognosis in AML with both inv(16) and t(8;21), and a higher incidence of relapse in AML with inv(16) (41–44).

Therefore, CBF-AML with *KIT* mutations has been reclassified into intermediate-risk group in the National Comprehensive Cancer Network recommendations (43). Nevertheless, data is still unclear since some authors attributed the negative prognostic effect of KIT alterations to only those present at an allelic burden higher than 25% or 35% (43, 45, 46).

Moreover, the prognostic effect of *KIT* mutation in pediatric CBF AML patients is still uncertain (47).

Given the prognostic implication of the gain-of-function *KIT* mutations, and over-expression of KIT observed in most CBF-AML including those with *KIT* mutations (48), studies have explored the addition of *KIT* inhibitors such as dasatinib and avapritinib to frontline therapy (49) to improve the outcome. Results are promising in terms of reducing relapse rates of *KIT* mutated CBF-AML to levels comparable to non-KIT mutated CBF AML (49) (**Tables 1**, **2** and **Figure 2**).

## WHO Classification Defining Genetic Mutations

### AML With Mutated *NPM1*

**Category**: DNA replication and cell cycle.

**Clinicopathologic and morphologic features**: monocytic/myelomonocytic phenotype; blasts with classical fish-mouth morphology.

**Recommended method of testing**: NGS, PCR followed by CE (50), ddPCR for serial monitoring (51).

*NPM1* is a molecular chaperone involved in cell cycle progression with multiple critical functions including ribosome biogenesis and transport, apoptotic response to stress stimuli, maintenance of genomic stability, and DNA repair (52). *NPM1* mutations are the most common genetic alteration in AML occurring in 25% to 41% of cases (53). These alterations affect exclusively the C-terminal region, leading to cytoplasmic mis-localization of the *NPM1* and leukemogenesis induction by inhibition of p53 activity (54). The most frequent mutation is a 4 base pair insertion of TCTG at position 956–959 (55). Multiple gene variants of NPM1 have been identified, all engendering the same biological effect. They are mostly associated with normal karyotype, and they are mutually exclusive with other known recurrent genetic abnormalities including *RUNX1* and *CEBPA.* Interestingly, *NPM1* mutations co-occur with mutations in epigenetic modifiers including DNMT3A, TET2 and IDH1/2 in 73% of the cases (55); these epigenetic alterations, unlike *NPM1*, are usually identified in pre-leukemic cells, and *NPM1* mutations are believed to be a later occurrence (56). *NPM1* mutated AML is considered a distinct biological subtype of AML in the latest (2016) WHO classification (57). In the settings of myelodysplastic syndrome (MDS) or myelodysplastic syndrome/myeloproliferative neoplasms (MDS/MPN), independent studies have suggested that the presence of *NPM1* mutation should be an AML-defining mutation, irrespective of the blast percentage, since these patients benefit from AML-type treatment with intensive chemotherapy (58, 59).

*NPM1* mutated AML is generally considered a favorable prognostic marker with good response to induction therapy (60). The prognosis is influenced by concurrent mutations in other genes particularly FLT3 allele ratio. AML patients with *NPM1* mutation with absent or low FLT3-ITD AR (<0.5) have similar OS, and are classified as favorable risk by 2017 ELN, whereas AML patients with mutated NPM1 and high FLT3 ITD AR are classified as intermediate-risk along with wild-type *NPM1* with absent or low FLT3 ITD AR. *NPM1* mutations in AML disappear with CR. However, the persistence of the mutation during follow-up is a sign of adverse prognosis (1). Unfortunately, residual leukemia is not always apparent by morphology or flow cytometry. Cocciardi et al. described the loss of *NPM1* mutation at relapse in 9% of *NPM1* mutated AML, a finding that alters the prognosis through the selection of clones that harbor FLT3 or DNMT3A mutations exclusively (61). Both of the above findings highlight the importance of serial NGS as a follow up tool for AML to identify the clonal evolution of the disease.

A significant improvement of OS was observed in *NPM1* mutated AML with Venetoclax, a BCL2 inhibitor, in combination with hypomethylating agents in patients > 65 years old (17). Venetoclax was approved by the FDA in combination with azacitidine or decitabine for the treatment of newly diagnosed AML in patients older than 75 years old or who have severe comorbidities (14, 18). Multiple other targeted therapeutic options for *NPM1* mutated AML are ongoing; these include: 1- Deguelin, a selective silencer of the *NPM1* mutation that stimulates apoptosis and induces differentiation in AML cells (62); 2- NSC34884 a molecula that disrupts the hydrophobic region that induce *NPM1* oligomerization leading to apoptosis; 3- CIGB-300 a molecule that binds *NPM1* to prevent the phosphorylation process resulting in induction of apoptosis (63); 4- Selinexor (KPT 330), an inhibitor of exportin 1 responsible of the cytoplasmic mis-localization of mutated *NPM1* (64); 6- EAPB0503, a molecule that promotes *NPM1* degradation and corrects the *NPM1* mis-localization causing an inhibition of the leukemia cell growth (65) (**Table 2**).

### AML With Mutated RUNX1

**Category**: myeloid transcription factor (22).

**Clinicopathologic and morphologic features:** minimally differentiated AML (AML-M0).

**Recommended method of testing**: NGS that includes the whole coding region (66).

*RUNX1* is a TF located on chromosome 21q22.12 recurrently involved in leukemia due to multiple types of alterations including chromosomal translocations, mutations, and copy number changes. Somatic mutations occur in up to 15% of the AML, and the frequency is higher in secondary AML arising from MDS (67). De novo AML with mutated *RUNX1* have characteristic clinicopathologic features that include male predominance, higher frequency of *SRSF2* and *ASXL1* mutations, normal karyotype, and absent NPM1 mutations (68).

Per 2017 ELN guidelines, mutated *RUNX1* is considered as an adverse prognostic factor. You et al. reported the results of a study on 219 patients with AML, those with *RUNX1* mutations had shorter relapse-free survival than patients with wild type-*RUNX1* (69). In a meta-analysis of four studies, *RUNX1* mutation was associated with dismal prognosis in AML (70). Stengel et al. reported a better outcome when *RUNX1* mutation was associated with IDH2 mutation, but worse when associated with ASXL1, SF3B1, SRSF2 and PHF6 mutations (71). Based on the poor outcome observed in studies that evaluated AML with *RUNX1* mutations, the 2016 revised WHO AML classification system regards de novo AML with mutated *RUNX1* as a provisional entity (57, 69, 70). However, when only de novo AML cases are evaluated, the outcome is similar to AML with wild-type *RUNX1* cases (68).

Notably, as *RUNX1* is a gene implicated in germline predisposition disorders, attention should be given to specific findings in *RUNX1* alterations. The presence of double mutations or mutations with near heterozygous or homozygous VAF, presence of mutation in familial clusters and history of thrombocytopenia should prompt investigation for an underlying familial platelet disorder with predisposition to myeloid malignancy (72).

While there are no direct targeted therapies at this time, enhancer suppression using bromodomain and extra-terminal motif (BET) inhibitor prevents aberrant *RUNX1* and *ERG* signal-induced transcription in pre-clinical studies (73). Using CRISPR/Cas9, the BET protein antagonist induced inhibition of *RUNX1* resulting in more apoptosis of leukemic cells expressing mutated *RUNX1* compared to wild-type cells, inducing an improvement of the survival of mice (74) (**Table 2**).

### AML With Mutated CEBPA

**Category**: myeloid TF.

**Clinicopathologic and morphologic features:** AML with or without maturation.

**Recommended method of testing**: NGS and PCR followed by direct sequencing (sanger-sequencing) (75).

CCAAT enhancer binding protein (*CEBPA)* is a TF located on chromosome 9q13.11 expressed in myeloid lineage. It plays a major role in proliferation and differentiation of the myeloid precursors to granulocytes or monocytes (76).

*CEBPA* mutations are found in 10–15% of patients (77). AML with biallelic *CEBPA* mutations in a heterozygous or homozygous pattern are significantly associated with better overall prognosis and outcomes independently of other molecular markers (77, 78). AML with single *CEBPA* mutations are uncommon, and more studies are needed to elucidate the clinical significance. Detection of biallelic *CEBPA* alterations should also prompt investigation for an underlying germline mutation on constitutional DNA with skin fibroblast culture and investigation of underlying familial predisposition (57, 79).

Due to the high GC content, *CEBPA* is a notoriously difficult gene to sequence. There are reports of successful CEBPA sequencing by NGS. This requires extensive modifications and tweaking of the PCR conditions and probe sequences (75).

Jakobsen et al. identified *NT5E* that encodes CD73 to be up-regulated in bi-allelic *CEBPA* mutant leukemia (80). The efficacy of CD73 inhibitors, including synergy with immune checkpoint inhibitors such as PD-1/PD-L1 are being evaluated as targeted therapy in bi-allelic *CEBPA* mutant AML (NCT03454451) (**Table 2**).

## Genes Involved in Prognostic Risk-Stratification of AML

### Tumor Protein p53 (*TP53)*

**Category**: tumor suppressor.

**Clinicopathologic and morphologic features**: associated with complex karyotype and shorter OS.

**Recommended method of testing**: NGS for detections mutations across the whole coding region and low mutant burden (81).

*TP53* is mutated in 8-14% of *de novo* AML cases, but as high as 73% in AML in older patients and therapy-related AML (26–28). The mutations induce loss-of-function, dominant negative and gain-of-function phenotypes (82). The presence of a *TP53* mutation is an independent predictor of poor survival and is associated with a high risk of recurrence and treatment resistance. The 2017 ELN classification regards the presence of *TP53* mutation as an adverse risk category (7).

One important additional information obtained from NGS for prognostication, beyond just the presence of a *TP53* mutation, is the variant allele frequency (VAF). In MDS, there is significant difference in prognosis between MDS with *TP53* VAF >40% *vs*. <20% (median OS of 124 months *vs*. OS not reached) (83). Prochazka et al. categorized 98 *de novo* AML cases using similar cut-offs: VAF >40%, VAF 20% - 40% and VAF <20%; sub-clonal *TP53* mutation (VAF <20%) showed a negative prognostic effect in terms of CR rate, OS and EFS (81). These findings highlight the importance of a sensitive molecular assay that detects minute sub-clones of TP53 mutation, and the importance of the genetic data provided by NGS, including accurate VAF.

The development of targeted therapy in *TP53* mutated cases should take into consideration multiple factors such as alterations affecting related pathways as well as other therapeutic options such as BCL2 inhibitors’ in the latter, the *TP53* activation may overcome resistance to BCL-2 inhibitors (84). Idasanutlin, an *MDM2* inhibitor (85) and Cobimetinib a MEK inhibitor (86), both affecting *TP53* expression, are being evaluated in combination with Venetoclax (BCL2 inhibitor) in phase I and phase II trials. Also, clinical trials exploring APR-246, a mutant P53 activator, are underway (NCT03931291); recently, in 2020, FDA granted breakthrough therapy designation for APR-246 in Combination with Azacitidine for the treatment of MDS with a *TP53* Mutation (87). The different roles of *TP53* in chemotherapy response and particularly the work of Zuber et al. on the activation of *TP53* gene in mice provide supportive data of the significant role of exogenous activation of *TP53* pathway in regard to treatment response (79) (**Table 2**).

## Gene Mutation Signatures for Identification of AML Ontogeny

Lindsley et al. evaluated the differences in gene mutation patterns between secondary AML, therapy-related AML, and *de novo* AML to decode the ontogeny of different subsets of AML. The authors identified a set of gene mutations that can provide an objective evidence of AML ontogeny irrespective of clinical information. The presence of a mutations in any of these genes: *SRSF2, SF3B1, U2AF1, ZRSR2, ASXL1, EZH2, BCOR*, or *STAG2* was >95% specific for the diagnosis of secondary AML (88). Majority of these genes, *SRSF2, SF3B1, U2AF1, ZRSR2*, are genes encoding proteins that belong to the spliceosomal complex that encompasses a large number of members including small nuclear ribonucleoproteins and protein factors responsible for removing introns from a transcribed pre-mRNA (89). *EZH2* and *BCOR* mutations are associated with worse OS in AML (55, 90, 91) while *STAG2* is a part of the cohesion complex.

The findings have major clinical and diagnostic implications. When detected in “*de novo*” setting, the presence of secondary AML mutations can identify a subset of patients with worse outcome (88). The “secondary-type” mutations, as expected, are frequently present in MDS (92) and chronic myelomonocytic leukemia (CMML) (93). These findings imply these mutations are unlikely to promote the development of acute leukemia without a co-operating event. It is also to be noted that these mutations occur early in the disease course and persist during follow-up despite morphologic CR, hence should not be used for MRD follow up purposes (**Figure 2** and **Table 2**).

## Uncovering Underlying Germline Mutations in AML and Familial Predisposition

Somatic NGS sequencing in AML can potentially uncover incidental germline mutations. The latest WHO recognizes myeloid neoplasms with germline predisposition syndromes as a distinct diagnostic category (57). Recognition of these conditions has major therapeutic implications (94) and different diagnostic and monitoring strategies for the patient himself, as well as the family members. When clinically suspicious, germline mutations testing and proper family screening are important, especially when an allogeneic bone marrow transplant is under consideration.

The new WHO classification introduced four broad categories of AML with germline predisposition: 1-nMyeloid neoplasms with germline predisposition without a pre-existing disorder or organ dysfunction, it includes AML with *CEBPA* and *DDX41* mutations 2- Myeloid neoplasms with germline predisposition and pre-existing platelet disorders, which encompass *RUNX1*, *ANKRD26* and *ETV6* mutations 3- Myeloid neoplasms with germline *GATA2* mutation and 4- Myeloid neoplasms with germline predisposition associated with inherited bone failure syndromes and telomere biology disorders. Hence, it is recommended to include these genes, *CEBPA, DDX41, RUNX1, ANKRD26, ETV6, TERT* and *GATA2*, within the standard NGS panel.

Since most of the mutated genes associated with germline predisposition disorders are also recurrently mutated in sporadic leukemia cases, attention should be given when interpreting the results of sequencing to identify clues suggesting a germline origin, such as double mutations, one with a near heterozygous or homozygous VAF (95). The presence of any of these mutations in a newly diagnosed leukemia is not sufficient to diagnose an AML with germline predisposition. Germline origin by testing constitutional DNA (skin fibroblast culture in most patients with active hematological malignancies) should be performed for confirmation (72, 88). It is important to highlight that targeted sequencing will not detect all the germline alterations such as intronic regions and large deletions spanning multiple exons; additional tests should be done to further investigate when clinically suspicious, such as array comparative hybridization for deletions and whole exome sequencing for novel mutations (96, 97). The confirmation of germline origin requires a prior genetic counseling since the results may cause significant disturbance of the affected individuals and families if not properly interpreted and handled (**Figure 2** and **Table 2**).

## Variant Allele Frequency of Gene Mutations Estimated by NGS Is Important for Treatment Decisions

NGS is a quantitative assay that provides information on mutant allele burden, which is critical for management decisions and predict outcome. VAF is defined as the ratio of reads with mutation against total (mutant + wild-type) reads. Hence, current NGS reports are generally not limited to presence or absence of mutations but include the VAF of each alteration. VAF is important for management decisions and outcome prediction in AML and MDS. This was previously elaborated in the context of TP53 mutations in AML and MDS (81, 83). A high mutant allele burden at diagnosis can be a negative prognostic factor. Patel et al. demonstrated a negative prognostic effect of high *NPM1* mutant allele burden at diagnosis in *de novo* AML cases (98). Sasaki et al. investigated the effect of the VAF of clonal hematopoiesis associated genes *ASXL1, DNMT3A, JAK2, TET2*, and *TP53* mutations on survival in 421 newly diagnosed AML using NGS. Higher VAF (cut-off of 30%) was associated with worse survival in AML patients within intermediate-risk cytogenetic group (99).

## Sequential NGS Assessment for MRD Chemoresistance and Early Relapse Detection

Serial NGS is ideal for monitoring AML patients for mutational clearance and/or clonal evolution and relapse. Clearance of somatic mutations in non-preleukemia genes at the time of CR was associated with better OS and decreased risk for relapse (100). Sequential analysis for persistent mutations is particularly helpful to evaluate residual disease in patients treated using novel targeted therapeutic agents, such as *IDH* inhibitors, as these can pose diagnostic challenges on morphology and/flow cytometry. Therefore, NGS is particularly useful to objectively evaluate evidence of residual disease in these circumstances.

The implications of NGS include assessment of effectiveness of therapy and detection of resistance mutations which can have implications on management and therapeutic regimen choices. Most importantly, sequencing of blast cells can detect *TP53* mutations which are independent predictors of poor survival and treatment resistance (7). Moreover, mutations that trigger resistance to *FLT3* inhibitors can be identified in AML cases including emergence of leukemic clones harboring mutations that activate RAS/MAPK pathway signaling (37). On the other hand, emergence of *FLT3*-TKD mutation during treatment can engender resistance to TKI; therefore, sequential mutational analysis is mandatory for early detection of potential treatment resistance and the choice of alternative drugs. Finally, the acquisition of *IDH1, WT1, ASXL1* variants in certain AML clones, either present at diagnosis or gained at relapse confer chemotherapy resistance (101).

While NGS facilitates identification of disease evolution and treatment resistance mutations, there are several caveats. First, the sensitivity of standard NGS panels used in clinical laboratories, currently limited to 2-5% VAF (102) is less or comparable to standard flow cytometry techniques for detection of residual disease at the time of morphologic remission. Yet, NGS results are more helpful than latter in settings where blasts show monocytic differentiation or in settings with limited sample quality. The identification of the mutations is partly limited by the intrinsic error rates (0.1% to 1%), that can be potentially overcome using the error correction methodologies such as molecular barcoding (103). Alternately, ultra-deep sequencing NGS with an ultra-high depth of coverage or individual gene assays such as ddPCR assays can be helpful (104). In one study, a high-throughput deep sequencing NGS method showed a detection sensitivity of 10^-4^ for SNVs and 10^-5^ for insertions/deletions (105).

Second, persistence of “pre-leukemia” mutation signature cannot be used for MRD detection. NGS enables the identification of background clonal hematopoiesis. Clonal hematopoiesis of indeterminate potential (CHIP), defined as the presence of a somatic alterations (either a somatic mutation associated with myeloid malignancy present at least 2% VAF or cytogenetic abnormality) in apparently healthy individuals. These patients have an increased risk of developing a hematologic malignancy, higher risk of developing therapy related myeloid neoplasms following chemotherapy for a solid tumor and increased frequency of adverse cardiovascular events (106).

Most of the CHIP alterations belong to genes *DNMT3A, TET2* and *ASXL1* (DTA) (106, 107). All three genes are involved in epigenetic regulation of myeloid differentiation and are considered to be within preleukemic hematopoetic stem cells (108). DTA gene mutations cannot induce leukemia without a co-operating second hits (109). The mutations in either one of the DTA genes impart negative prognostic outcomes in AML (110–112). The 2017 ELN classifies *ASXL1* mutations as adverse risk category (7), and its presence was >95% specific for AML of secondary origin (88).

Mutations in all three genes can be seen within the entire coding region, and include missense, frameshift, nonsense and splice-site mutations leading to a non-functional protein. The only exception being *DNMT3A*, which has a hotspot point mutation in codon R882 (113, 114). Loss of *TET2* function can also occur *via* mutations in *IDH1*, *IDH2* and *WT1* (115, 116), that explain the mutual exclusivity between *IDH1/2-TET2-WT1* mutations in AML (116). Hence, NGS is an ideal technique for detection of all these mutations.

Importantly, DTA gene mutations likely persist at the time of remission of AML in pre-leukemic clones, hence they cannot be used to detect MRD (109). *DNMT3A* R882 mutation persists in 75% of AML patients during remission without any negative impact on outcomes (117). Interestingly, ascorbate supplementation can restore methylation patterns and minimize proliferation of blasts (118). A clinical trial evaluating the efficacy of azacitidine and high dose ascorbic acid in AML with mutated *TET2* is ongoing (NCT03397173).

9- Subclonal evolution in AML using single-cell technology:

All malignancies are genetically heterogeneous, composed of mutationally-defined subclonal cell populations characterized by distinct phenotypes. Precise identification of clonal and sub-clonal architecture is mandatory to understand the temporal evolution of tumor and the emergence of treatment resistance. Bulk sequencing cannot definitively resolve the actual complex clonal composition of neoplasms generally and AML specifically. Therefore, there is great interest in understanding the genetic alterations at a single-cell level using the newly designed sequencing platforms. Multiple reports published recently highlighted the subclonal selection during the treatment journey. Morita et al. reported the sub-clonal complexity in 37, clonal architecture and mutational histories of 123 AML patients. The authors explored single cell-level mutation co-occurrence and mutual exclusivity revealing novel clonal relationships; and emergence and selection of resistant subclones under therapies by longitudinal analysis (119). Interestingly, current emerging single-cell multi-omics technology, aid in profiling simultaneously single-cell mutations and cell surface proteins in AML cases, allowing correlation of genetic and phenotypic heterogeneity (120). Using the novel features of this technology, Petti et al., using a high-throughput platform to distinguish tumor and non-tumor cells in AML, identified tumor cells showing phenotypic aberrancies and lineage infidelity, evaluated the sub-clonal progression of tumor samples with time with a molecular signature for each sample, and cell-surface markers that could be used to isolate specific cells for downstream studies (121). Another study showed that *FLT3*-ITD mutation was present in the primitive cells, whereas *FLT3*-TKD mutation was present in the more differentiated cells within the same tumor. The study demonstrated that *FLT3* variants differentially affected AML differentiation that explained the worse prognosis associated with certain alleles (122).

## Discussion

We believe that NGS is an exciting tool that has helped pathologists and oncologists tremendously to improve their understanding of AML pathogenesis and clonal evolution of the disease. It has played a major role in designing therapies targeting the disease and control relapse. Genomic analysis of cases at diagnosis and relapse have uncovered the alterations and clonal evolution of the genetic profile of the tumor cells during disease progression. The cited studies and clinical trial results highlight the unique genetic signature of every patient’s disease, not only with respect to different combinations of mutations, but also in terms of clonal burden of different mutations, sequence of mutations, and concurrent chromosomal gain and losses. As a consequence of this, 1- the prognostic outcome of each patient can be unique and cannot be simply generalized based on the presence or absence of mutations; 2- the therapeutic molecules should be targeted to attack both the primary clone and emerging sub-clones with potential resistance mechanisms. Hence, a better understanding of each patient’s AML genome by NGS is mandatory to make decisions related to appropriate personalized therapy.

Nevertheless, a lot more standardization is needed for implementing NGS in daily clinical practice; for instance, so far there is no universal consensus, yet on which target genes should be included in the sequencing panels. Moreover, the sequencing depth of coverage is still a subjective number chosen by each laboratory for the validation of their sequencer. Furthermore, there is no consistent practice for quality assessment of sequencing data; the accuracy of results is compromised at genome locations with highly repetitive sequences, or in GC-rich locations, common problems encountered during sequencing and data analysis. Finally, results interpretation can be time-consuming and requires specific expertise from bioinformatics and pathology (or equivalent degree). Further standardizations are needed when NGS is used for serial follow-up to assess measurable residual disease. It is important to keep in mind that bulk NGS represents the “average” findings per cell, and hence the data on genomic complexity is only inferred. On the other hand, single-cell NGS can accurately provide concrete information on sub-clonal architecture, but it has not reached the mainstream clinical work-flow yet (123).

At the same time, while NGS is an important component of AML workup, other testing needs to be performed to accurately sub-classify the disease, specify the prognosis, and determine the best targeted therapy. A comprehensive workup for AML should include a karyotype to identify the disease defining chromosomal translocations including t(8;21), t(15;17), t(16;16) and the new WHO entity AML with *BCR/ABL* (57); however, some fusions can be cryptic (57), and other testing is required to highlight these aberrations including Fluorescence in situ hybridization (FISH), RT-PCR, RNAseq, and other targeted fusions assays. Moreover copy-neutral loss of heterozygosity (cnLOH) has prognostic significance in patients with acute leukemia (124); Walker et al. showed that LOH mediated by uniparental disomy (UPD) is a common finding in cytogenetically normal AML. Also, UPD involving 13q and 11p are important for genetic risk stratification in these cases (125). FISH and karyotyping can be used to depict copy number aberrations; however, they are limited by low resolution or restriction to targeted assessment. The alternative method of testing is Chromosomal microarray which can characterize chromosomal copy number changes and cnLOH in myeloid malignancies (126).

Overall, NGS has enabled phenomenal advances in understanding of molecular genetics of AML and opened up new horizons for development of highly effective therapeutic molecules and protocols for individualized treatment and monitoring that are completely reshaping the management of the different subtypes of AML.

## Author Contributions

All authors contributed to writing the manuscript, reviewing the final version and preparing the figures and tables. All authors contributed to the article and approved the submitted version.

## Conflict of Interest

The authors declare that the research was conducted in the absence of any commercial or financial relationships that could be construed as a potential conflict of interest.

## Publisher’s Note

All claims expressed in this article are solely those of the authors and do not necessarily represent those of their affiliated organizations, or those of the publisher, the editors and the reviewers. Any product that may be evaluated in this article, or claim that may be made by its manufacturer, is not guaranteed or endorsed by the publisher.
